# Insecticide Exposure and Risk of Asthmatic Symptoms: A Systematic Review and Meta-Analysis

**DOI:** 10.3390/toxics9090228

**Published:** 2021-09-21

**Authors:** Jiraporn Chittrakul, Ratana Sapbamrer, Wachiranun Sirikul

**Affiliations:** Department of Community Medicine, Faculty of Medicine, Chiang Mai University, Chiang Mai 50200, Thailand; jerasooutch@gmail.com (J.C.); wachiranun.sir@gmail.com (W.S.)

**Keywords:** insecticide, asthmatic symptoms, farmer

## Abstract

The incidence of respiratory disease is increasing. In relation to this, in addition to infection, factors associated with working with chemical insecticides are a cause for concern. Some of the chemicals involved have been shown to affect the respiratory system, and consequentially workers are at increased risk of conditions such as asthma. However, medical opinion around this area is still controversial; therefore, the objective of this study is to investigate the association between exposure to insecticides and asthma by means of a systematic review of the relevant literature. Relevant literature was identified, and a systematic review was conducted to investigate the association between exposure to insecticides and asthma. A total of five studies (three cross sectional and two cohort) including 45,435 subjects were identified as relevant. The summary odds ratios related to the impact of exposure to specific insecticides on asthma were organophosphates 1.31 (95%CI = 1.17–1.48, I^2^ = 27%, *p* = 0.172), carbamates 1.44 (95%CI 1.08–1.92, I^2^ = 56.7%, *p* = 0.031) and organochlorines 1.31 (95%CI 1.19–1.64, I^2^ = 37.3%, *p* = 0.131). Farmers exposed to certain insecticides may have an increased risk of asthma and asthmatic symptoms, but further research on that issue is urgently needed.

## 1. Introduction

Chronic respiratory disease and asthma are major public health issues that cause physical and psychological morbidity and mortality [1]. They also affect the medical and societal costs of patients. The World Health Organization (WHO) has stated that chronic respiratory disease causes the death of approximately >3 million people, and a further 235 million suffer from asthma [2]. There are several risk factors for asthma, including genetics, indoor allergens, respiratory viruses, air pollution, volatile organic compounds, and pesticides [1,3]. The WHO has identified pesticide poisoning as a major public health problem that causes significant morbidity and mortality worldwide [4]. Pesticides are a group of chemicals that are classified according to their target organisms, specifically insecticides, herbicides, and fungicides [5]. Insecticides, for example, are chemicals that kill and repel insects, and, classified by their chemical nature, the most common include: organophosphates, carbamates, organochlorine, pyrethroid, and neonicotinoids [6,7,8]. The most common chemicals in insecticides used in agriculture are organophosphates, carbamates, and organochlorine, whereas pyrethroid is commonly used for household and public health purposes [9]. Exposure to insecticides might cause both acute and chronic adverse effects [10,11]. Long-term exposure to insecticides can have chronic effects including certain types of cancer, Parkinson’s disease, Alzheimer’s disease, multiple sclerosis, diabetes, coronary heart disease, chronic kidney disease, and respiratory diseases [11,12,13]. Occupational exposure to pesticides is common for farmers, farm workers, and industrial workers involved in pesticide production [12,13]. They can be exposed to the pesticides via three common routes: through the skin (contact), the mouth (ingestion), and the lungs (inhalation) [14]. The inhalation of insecticides can cause serious damage to the nose, the throat, and lung tissues, causing inflammation of the airways and frequently leading to chronic respiratory disease [15]. According to previous studies, occupational exposure to insecticides is associated with an increased risk of the respiratory symptoms common in asthma and chronic bronchitis [16]. However, some studies have not found any association between insecticide exposure and asthma [17]. Therefore, a review of all of these studies needed to be conducted to produce a meta-analysis of this important area. The findings of this study will provide powerful evidence in support of the hypothesis that insecticide exposure is related to an increased risk of asthma.

## 2. Materials and Methods

### 2.1. Searching Strategy

This study was conducted in accordance with the systematic literature review and meta-analysis reporting guidelines of the Preferred Reporting Items for Systematic Re-views and Meta-Analysis (PRISMA). The current studied is registered under PROSPERO registration number: CRD42021270516, 28 August 2021. The team searched for relevant articles published in the following databases: Web of Science, Scopus, PubMed, Google Scholar, and Google, using the following keywords: “non-atopic asthma” OR “atopic asthma” OR “asthma” plus “pesticide” OR “Insecticides”.

### 2.2. Inclusion Criteria

The articles that were included were the ones that met the following criteria: (1) original article; (2) published in a journal or thesis; (3) published as a full article; (4) published between 1990 and 2021; (5) assessed the effect of pesticide use on asthma in farmers; (6) occupational exposure; (7) written in English; and (8) data analyzed by correlation coefficient analysis, regression analysis, or discriminant analysis to identify the direction of association by adjusting any interference factors. The exclusion criteria were as follows: (1) articles without variables of interest; (2) review articles; (3) articles with irrelevant information.

### 2.3. Data Extraction

The data were extracted from the identified articles by the name of the first author, publication year, country, study design, number of the population, age, gender, length of work, diagnosis of asthma, name of the chemical, adjusted odds ratio (OR), and confounding variables. The adjusted OR is the result of estimating the association between insecticide exposure with asthma risk in farmers. Two investigators extracted the data independently.

### 2.4. Quality Assessment

We assessed the quality of each article using the National, Heart, Lung and Blood Institute (NIH) statement: Guidelines for Reporting Observational Studies. https://www.nhlbi.nih.gov/health-topics/study-quality-assessment-tools (accessed on 7 June 2021). The NIH checklist consists of 14 items for assessing the quality of the selected observational studies. Two reviewers (RS and JC) independently assessed the quality of reporting in each study. Assessment criteria require reviewers to rate studies to assess the risk of bias in the study due to defects in study design or execution. Ratings are given for a range of items included in each tool to judge each study, with the quality being categorized as “good,” “fair,” or “poor”. The first criterion, “good” means there is the least bias. The second criterion, “fair,” is susceptible to some bias, but the level is considered insufficient to invalidate the results. The final score of “poor” indicates a risk of study bias. (Table S1: available as Supplementary data).

### 2.5. Statistical Analysis

The criterion of interest was occupational exposure to insecticides, including organophosphates, carbamates, and organochlorines. The outcome of interest was reported asthmatic symptoms by the patient or diagnosis by a medical doctor. From the eligible studies, adjusted odds ratios (aORs) with 95% confidence intervals (95%CIs) were retrieved and were utilized as a summary measure for a meta-analysis of the study results. The pooled estimates of the relationship between exposure to insecticides and asthmatic symptoms were analyzed using a fixed-effect model utilizing the Mantel–Haenszel method and a random-effect model using the DerSiomonian and Laird method. To assess heterogeneity, we used the Cochran Q and I^2^ tests against each other. We determined heterogeneity using the value of I^2^. An I^2^ value < 25% equated to low heterogeneity; I^2^ 25–50% indicated moderate heterogeneity; and I^2^ > 50% indicated large heterogeneity. Funnel plots displaying the log OR of individual studies on the horizontal axis and standard error on the vertical axis were used to detect potential bias from small study effects (e.g., publication bias). All of the statistical tests were two-tailed, and *p* <0.05 was used to denote statistical significance. Quantitative synthesis of the data and all of the statistical analyses were performed using the STATA software package (Stata Corp. 2019. Stata Statistical Software: Release 16. College Station, TX, USA: Stata Corp LLC).

## 3. Results

### 3.1. Search Study

**Figure 1:**
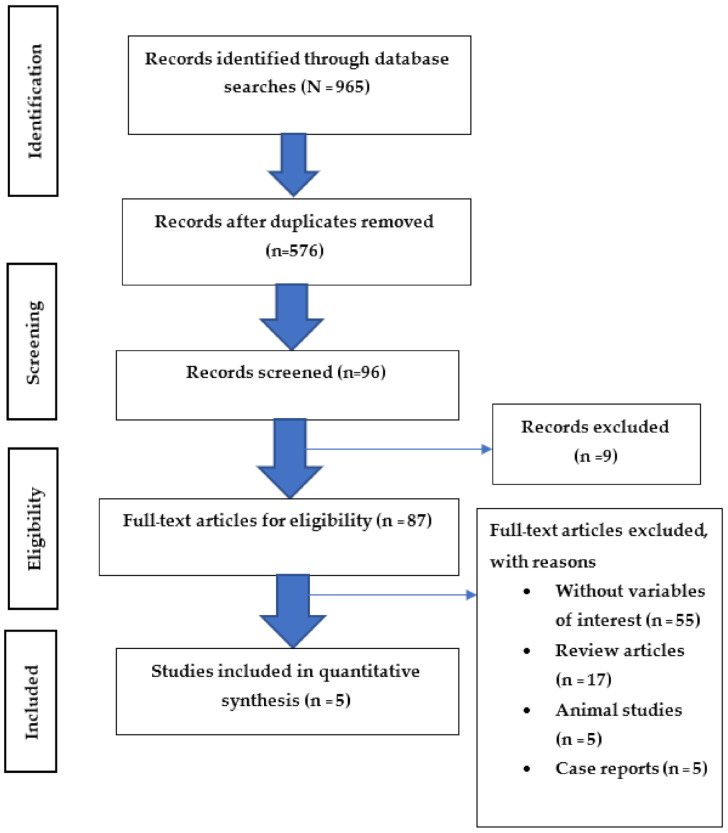
Flow chart of the study selection process (PRISMA). N = records identified through database searches, n = records after database searches.

The flow diagram in Figure 1 shows a summary of the method that was used. Our initial search of all of the databases retrieved 965 studies. After duplicates were removed, 576 articles remained, and 96 articles were screened based on the title and/or the abstract to determine eligibility. After screening, nine articles were excluded because there was no full-text paper. There were 87 full-test articles that were eligible. Eighty-two articles were excluded for the following reasons: (1) they were studies with no variables of interest (environmental exposure, biochemical studies, non-specific pesticides and other pesticides which were not insecticides, and exacerbation of asthma); (2) they were review articles; (3) they were animal studies; (4) they were case reports. Therefore, five studies were included in the quantitative synthesis.

### 3.2. Study Characteristics

The five studies identified for inclusion in the study included a total of 45,435 subjects [17,18,19,20,21]. The characteristics of the studies included in the meta-analysis are summarized in Table 1, Table 2 and Table 3. Using the inclusion criteria described in the method, five articles were identified, and all of them were studies reporting on investigations into insecticide exposure that resulted in asthma disease. Three studies were cross-sectional, and two were prospective cohort studies. All of the studies focused on pesticides and their effects on farmers resulting in a diagnosis of asthma, with the biggest impact on farmers being from insecticide pesticides. The average age of the participants was only mentioned in two studies. Senthilselvan et al. and Faria et al. [17,18] reported an average age of the cohort (mean ± SD), 45.2 ± 15.6 and 42 ± 15.6 years, respectively. Three studies included both genders, while two studies included only males. Geographically, four studies were performed in North America (Canada and the USA). A single study by Faria et al. [17] was conducted in South America (Brazil).

All of the studies in rural and farming areas included occupational exposure. In four studies, the reviewers collected information using a structured questionnaire [17,18,19,21]. One study used a telephone interview technique for data collection [20]. Regarding asthma screening, four studies used a questionnaire to collect self-reported data for the diagnosis of asthma, for example: “Has a doctor ever told you that you have asthma?” [17,18,20,21]. The fifth study was defined as a medical doctor’s diagnosis of asthma [19].

### 3.3. Insecticide Exposure and Possible Risk of Asthmatic Symptoms

Hoppin et al. (2009) [19] found that asthma was significantly associated with the use of coumaphos, diazinon, and parathion (2.34, 95%CI: 1.49 to 3.7, 1.57, 95%CI: 1.05 to 2.35 and 2.05, 95%CI: 1.21 to 3.46). Four studies, on the other hand, found that the use of organophosphates was not significantly associated with asthma (Table 1).

Senthilselvan et al. (1992) [18] found the prevalence of asthma was significantly associated with the use of carbamate insecticides (prevalence odds ratio = 1.8, 95%CI: 1.1 to 3.1). Their findings were consistent with the paper by Patel et al. (2018) [21], which reported that asthma was significantly associated with the use of carbaryl insecticides (2.3, 95%CI: 1.4 to 3.7). In contrast, two studies [19,20] found that the use of carbamate insecticides did not significantly increase the risk of asthma (Table 2).

Two studies [18,19] found that exposure to organochlorine insecticides was associated with asthma. One study found that asthma was statistically associated with the use of organochlorines [18]. Hoppin et al. (2009) [19] found that asthma was significantly associated with the use of chlordane, heptachlor, and lindane (1.77, 95%CI: 1.19 to 2.63, 2.01, 95%CI: 1.3 to 3.11 and 1.57, 95%CI: 1.01 to 2.41), respectively (Table 3).

### 3.4. Meta-Analysis of Exposure to Insecticides and Asthma Disease

**Figure 2:**
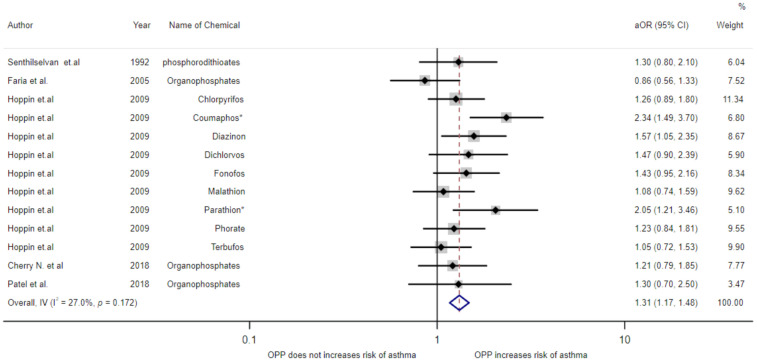
Meta-analysis of exposure to organophosphates and asthma disease. * *p* < 0.05.

The multivariable-adjusted ORs of exposure to organophosphates from all of the studies are shown in Figure 2. The pooled OR estimates found that exposure to organophosphates was associated with an increased risk of asthmatic symptoms (OR = 1.31, 95%CI 1.17–1.48). Statistically significant heterogeneity was detected (I^2^ = 27%, *p* = 0.172).

**Figure 3:**
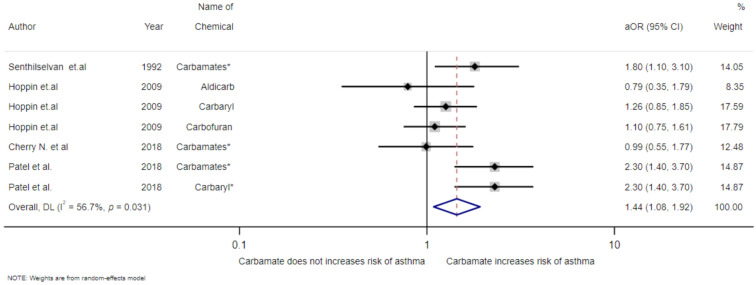
Meta-analysis of exposure to carbamates and asthma disease. * *p* < 0.05.

The multivariable adjusted ORs of exposure to carbamates from all studies are shown in Figure 3. The pooled OR estimates found that carbamate exposure was associated with an increased risk of asthmatic symptoms (OR = 1.44, 95%CI 1.08–1.92). Statistically significant heterogeneity was detected (I^2^ = 56.7%, *p* = 0.031).

**Figure 4:**
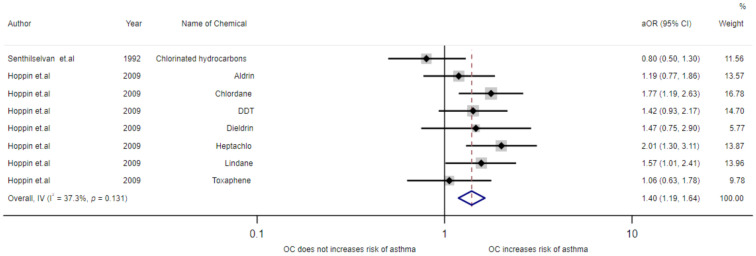
Meta-analysis of exposure to organochlorines and asthma disease.

The multivariable-adjusted ORs of exposure to organochlorines from all studies are shown in Figure 4. The pooled OR estimates found that organochlorines exposure was associated with an increased risk of asthmatic symptoms (OR = 1.31, 95%CI 1.19–1.64). Statistically significant heterogeneity was detected (I^2^ = 37.3%, *p* = 0.131).

### 3.5. Funnel Plot

**Figure 5:**
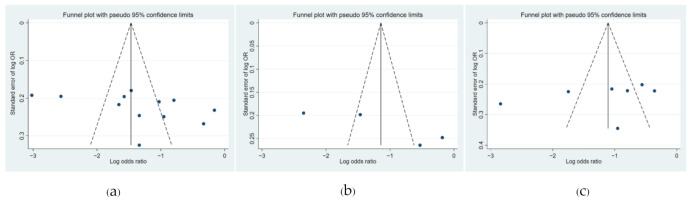
Funnel plot; (**a**) organophosphates; (**b**) carbamates; (**c**) organochlorines.

The funnel plots showed an asymmetrical funnel plot from the overall analysis, which may be because the size of effect differs according to study size, but there is little location bias (Figure 5).

## 4. Discussion

The existence of an association between insecticides and asthma was systematically assessed in this study using a meta-analytical approach. The relationship between insecticide exposure and risk of asthma has generated an increasing amount of interest in recent years. The results of our meta-analysis showed a significant increase in the risk of asthma among subjects with a history of insecticide exposure. The overall OR was 1.44, 95%CI 1.08–1.92, with an OR = 1.31, 95%CI 1.17–1.48, and OR = 1.31, 95%CI 1.19–1.64 in carbamates, organophosphates, and organochlorines, respectively. One previous study investigated the relationship between non-specific pesticides and the risk of asthma. Beard et al. (2003) [22] conducted a prospective cohort study that showed that occupational exposure to pesticides was associated with asthma (OR = 1.59, 95%CI: 1.05–2.43). Similarly, Stoecklin-Marois et al. (2015) [23] conducted a community-based cohort study regarding occupational exposure of pesticides and found that working in agriculture for years was associated with asthma (OR =1.04, 95%CI: 1.00–1.09). In the case of females who worked 15 years or more, OR = 3.60 (95%CI: 1.16–11.16), which shows that they have an increased risk of developing asthma. In addition, a cross-sectional study by Arroyo et al. (2018) [24] found that exposure to pesticides in the last 12 months was associated with asthma (OR = 1.55, 95%CI: 1.10–2.18). With regard to any association between herbicides and asthma, a study by Diaz-Criollo et al. (2020) [16] showed that paraquat was associated with self-reported asthma with a prevalence rate of 1.06 (1.00–1.13). A study of Cha et al. (2012) [25] also found the association between paraquat application and adverse respiratory health effects among farmers.

Some studies describe the mechanisms involved in the aggravate of asthma as a result of pesticide use. One example is that direct exposure to chemical pesticides, especially insecticides, will stimulate the bronchial tubes to cause inflammation in the immune system and will increase allergens in the body [26]. Pesticides have also been found to cause damage to DNA and to trigger cell apoptosis via their genotoxic effects [26,27]. Insecticides containing organophosphate chemicals are commonly used for agriculture, gardening, and household purposes, and these and other similar chemicals can become residual in the environment [28]. These chemicals are known to be acutely toxic to higher vertebrates, and if they enter the body, they are distributed to the organs [28]. In addition, some of these chemical residues are toxic to the body, causing the inhibition of the enzyme acetylcholinesterase, an important neurotransmitter. The inhibition of acetylcholinesterase will result in the inability to decompose acetylcholine. Consequently, acetylcholine accumulates in the synaptic gaps in the brain and at the nerve–muscle junctions. This results in the excessive activation of the muscarinic, and the nicotinic receptors by the above mechanisms causes disorders in the functioning of the central nervous system, peripheral nervous system, and autonomic nervous system, showing symptoms such as bronchospasm/dilation, etc. [29,30,31]. As the parasympathetic system is part of the autonomic nervous system where acetylcholine acts as neurotransmitter, the contraction of the smooth muscles in the airways may be affected by acetylcholine overflow. Hence, bronchospasm may occur, which is associated with asthma. In addition, the inflammation of the airways is more probable due to those chemicals [29,30,31]. Furthermore, a previous study discovered that M2 receptor dysfunction induced by organophosphates causes asthma in humans [26,32].

The occurrence of asthma could also be explained by the neurogenic inflammation caused by pesticide inhalation, the chemicals irritating sensory C-fibers in the airways, providing the release of vasoactive sensory neuropeptides [15,33]. Thus, pesticide droplets or vapors can damage the airways. Direct irritation of the mucous membranes of the bronchi leads to the inflammation of the airways. This activates the TRPV1 and TRPA1 ion channels in the bronchi. C-sensory fibers, inflammatory cells, and subsequently epithelial cells may release inflammatory neuropeptides. If airway inflammation persists for any length of time, bronchial hyperreactivity may occur, leading to asthma symptoms [15,33]. A previous study found that pesticides can cause oxidative stress, which leads to the release of free radicals and lipid peroxidation. Organophosphates and organochlorines are associated with an increase in reactive oxygen species and lipid peroxidation, a reaction between free radicals and unsaturated fatty acids in the cell membrane [34,35]. When injury occurs, damage also occurs in the cell membrane, impairing its function and role, which affects the signaling products of lipid peroxidation, such as HNE [36,37]. The oxidative stress caused by the pesticides also results in protein oxidation, a reaction between free radicals and proteins. These changes affect the function of enzymes and signaling molecules and may cause DNA oxidation, which affects both the integrity and expression of the genes, leading to possible transcription errors. As a result of all of these changes, the cells and tissues suffer from injuries, which result in inflammation [36,37]. In addition to these, other previous studies have also discovered that oxidative stress plays a role in inflammatory diseases, including those affecting the respiratory system [34,35,36,37].

Our study is a comprehensive meta-analysis investigating a possible link between insecticide exposure and the risk of asthma. However, the sensitivity level of the analysis of the study did not add weight to the substantiation of the association between insecticide exposure and the risk of asthmatic symptoms, indicating instability in the results. Therefore, more studies are needed.

## 5. Conclusions

The results of this systematic review and meta-analysis suggest that insecticides are associated with the risk of asthma. Exposure to organophosphates, carbamates, and organochlorines is closely related to asthmatic symptoms. Long-term occupational exposure to pesticides is associated with a diagnosis of asthma and asthmatic symptoms. However, the impact of the concentration of pesticides concerning the relationship to asthmatic symptoms is unclear. These findings provide powerful evidence in support of the hypothesis that insecticide exposure is related to an increased risk of asthma. Further research should be conducted to confirm the findings in our study and to clarify the mechanisms involved by more high-quality and large-sample case-controlled and cohort studies.

## Figures and Tables

**Table 1:** The characteristics of studies evaluating the association between exposure to organophosphates and asthmatic symptoms.

Authors (Years)/ Country	Study Design	Age Mean ± SD (Years)	Gender	Sample Size	Length of Work	Diagnosis of Asthma	Name of Chemical	Adjusted OR (95%CI)	Confounding Variables
Senthilselvan et al. (1992)/Canada [18]	Cross-sectional	45.2 ± 15.6	Both genders	2375	-	Self-report	Phosphorodithioates	1.3(0.8–2.1)	Age, height, weight, smoking pack-years
Faria et al. (2005)/Brazil [17]	Cross-sectional	42 ± 15.6	Both genders	1379	-	Self-report	Organophosphates	0.86(0.56–1.33)	Sex, age, schooling, marital status, smoking, socioeconomic indicators, agricultural production, exposure to dust, industrial rations, years of chemical exposure
Hoppin et al. (2009)/USA [19]	Cohort	-	Male	19,704	≥31 years	Use genitive in doctor’s diagnosis	Chlorpyrifos Coumaphos Diazinon Dichlorvos Fonofos Malathion Parathion Phorate Terbufos	1.26(0.89–1.80) 2.34(1.49–3.70) 1.57(1.05–2.35) 1.47(0.90–2.39) 1.43(0.95–2.16) 1.08(0.74–1.59) 2.05(1.21–3.46) 1.23(0.84–1.81) 1.05(0.72–1.53)	Age, smoking, state, high pesticide exposure events, and BMI
Cherry et al. (2018)/ Canada [20]	Cohort	-	Male	10,767	0–≥35 years	Self-report	Organophosphates	1.21(0.79–1.85)	Sex, age, smoking, work state now, exposure to pesticide in last month, ever had symptoms of pesticide poisoning, doctor has said you have allergies, doctor has said you have asthma
Patel et al. (2018)/USA [21]	Cross-sectional	-	Both gender	11,210	-	Self-report	Organophosphates	1.3(0.7–2.5)	Sex and region

**Table 2:** The characteristics of studies evaluating the association between exposure to carbamates and asthmatic symptom.

Authors (Years)/ Country	Study Design	Age (Mean ± SD) (Years)	Gender	Sample Size	Length of Work	Diagnosis of Asthma	Name of Chemical	Adjusted OR (95%CI)	Confounding Variables
Senthilselvan et al. (1992)/Canada [18]	Cross-sectional	45.2 ± 15.6	Both genders	2375	-	Self-report	Carbamates	1.8(1.1–3.1)	Age, height, weight, pack-years
Hoppin et al. (2009)/USA [19]	Cohort	-	Male	19,704	≥31 years	Use genitive in doctor’s diagnosis	Aldicarb Carbaryl Carbofuran	0.79(0.35–1.79) 1.26(0.85–1.85) 1.10(0.75–1.61)	Age, smoking, state, high pesticide exposure events, and BMI
Cherry et al. (2018)/ Canada [20]	Cohort	-	Male	10,767	0- ≥35 years	Self-report	Carbamates	0.99(0.55–1.77)	Sex, age, smoking, work state now, exposure to pesticide in last month, ever had symptoms of pesticide poisoning, doctor has said you have allergies, doctor has said you have asthma
Patel et al. (2018)/USA [21]	Cross-sectional	-	Both gender	11,210	-	Self-report	Carbamates Carbaryl	2.3(1.4–3.7) 2.3(1.4–3.7)	Sex and region

**Table 3:** The characteristics of studies evaluating the association between exposure to organochlorines and asthmatic symptom.

Authors (Years)/ Country	Study Design	Age (Mean ± SD) (Years)	Gender	Sample Size	Length of Work	Diagnosis of Asthma	Name of Chemical	Adjusted OR (95%CI)	Confounding Variables
Senthilselvan et al. (1992)/Canada [18]	Cross-sectional	45.2 ± 15.6	Both genders	2375	-	Self-report	Chlorinated hydrocarbons	0.8(0.5–1.3)	Age, height, weight, pack-years
Hoppin et al. (2009)/USA [19]	Cohort	-	Male	19,704	≥31 years	Use genitive in doctor’s diagnosis	Aldrin Chlordane DDT Dieldrin Heptachlor Lindane Toxaphene	1.19(0.77–1.86) 1.77(1.19–2.63) 1.42(0.93–2.17) 1.47(0.75–2.90) 2.01(1.30–3.11) 1.57(1.01–2.41) 1.06(0.63–1.78)	Age, smoking, state, high pesticide exposure events, and BMI

## Data Availability

Not applicable.

## Supplementary Materials

The following are available online at https://www.mdpi.com/article/10.3390/toxics9090228/s1, Table S1: The National Heart, Lung and Blood Institute (NIH) for cohort studies and cross-sectional studies.
