# Supplementary material for: Insecticide Exposure and Risk of Asthmatic Symptoms: A Systematic Review and Meta-Analysis
Source: Toxics. 2021 Sep 21;9(9):228. doi: 10.3390/toxics9090228 (PMC8473102; doi:10.3390/toxics9090228)
Supplement: Supplementary file 1 [file toxics-09-00228-s001.zip › toxics-1387239-supplementary.pdf]

toxics

Supplementary Materials: Insecticide Exposure and Risk of Asthmatic Symptoms: A Systematic Review and Meta-Analysis

Jiraporn Chittrakul, Ratana Sapbamrer * and Wachiranun Sirikul

Table S1. The National, Heart, Lung and Blood Institute (NIH) criteria for cohort and cross-sectional studies.

Study	Criteria														Total
	1	2	3	4	5	6	7	8	9	10	11	12	13	14	
**Cohort studies**															
Hoppin et.al	yes	yes	no	yes	yes	yes	yes	yes	yes	no	yes	yes	yes	yes	good
Cherry N. et al.	yes	yes	no	yes	yes	yes	yes	yes	yes	yes	yes	yes	yes	yes	good
**Cross-sectional studies**															
Senthilselvan et.al	yes	yes	yes	yes	yes	yes	yes	yes	yes	no	yes	CD	CD	yes	good
Faria et al.	yes	yes	yes	yes	yes	yes	yes	yes	yes	no	yes	CD	CD	yes	good
Patel et al.	yes	yes	no	yes	yes	yes	yes	yes	yes	no	yes	CD	CD	yes	good
